# The Real-World Study of Immunogenicity and Safety of the Adjuvant Recombinant Vaccine against Varicella Zoster Virus in Patients with Immune-Mediated Inflammatory Diseases Treated with Janus Kinase Inhibitors

**DOI:** 10.3390/vaccines11101610

**Published:** 2023-10-18

**Authors:** Ana Esteban-Vazquez, Martina Steiner, Elisabet Castañeda, Cristina Andreu-Vazquez, Israel J. Thiussard, Angela Somodevilla, Moisés Gracia-Martínez, Rosa Sánchez-Diaz, Cristina García-Yubero, Maria Beatriz Paredes-Romero, Santiago Munoz-Fernández

**Affiliations:** 1 Rheumatology Department, Infanta Sofía University Hospital, 28703 Madrid, Spain; martina.steiner@salud.madrid.org (M.S.); elisabet2993@gmail.com (E.C.); moises.gracia@salud.madrid.org (M.G.-M.); rosamasandi@yahoo.es (R.S.-D.); maritapr84@gmail.com (M.B.P.-R.); santiago.munoz@salud.madrid.org (S.M.-F.); 2 Statistics Deparment, Universidad Europea, 28703 Madrid, Spain; cristina.andreu@universidadeuropea.es (C.A.-V.); israeljohn.thuissard@universidadeuropea.es (I.J.T.); 3 Infanta Sofia University Hospital and Henares University Hospital Foundation for Biomedical Research and Innovation (FIIB HUIS HHEN), 28702 Madrid, Spain; 4 Microbiology Department, UR Salud Laboratory, 28703 Madrid, Spain; asomodevilla@ursalud.com; 5 Pharmacology Department, Infanta Sofía University Hospital, 28703 Madrid, Spain; cristina.gyubero@salud.madrid.org

**Keywords:** herpes zoster, varicella zoster vaccines, Janus kinase inhibitors, rheumatic diseases, Spain

## Abstract

**Background**. The risk of herpes zoster reactivation is increased in immunocompromised patients, especially in those with immune-mediated inflammatory diseases (IMIDs) on Janus kinase inhibitor (JAKi) treatment. The recombinant subunit herpes zoster vaccine (RZV) is a non-live vaccine, recently approved for this subgroup of patients, which shows high rates of vaccine effectiveness, with few adverse effects reported in clinical trials. **Purpose**. The aim of this real-world study was to determine the immunogenicity and safety of RZV in IMID patients on JAKi treatment. **Methods**. The increase in the concentration of anti-gE antibody for varicella zoster virus post-vaccination, compared to the pre-vaccination concentration, was analyzed to test the humoral immune response. Adverse effects after the first and second vaccine doses were registered. **Results**. In total, 49 patients were analyzed, and a fourfold increase in antibody concentration was achieved in almost 40% of subjects, with only one serious local adverse effect. **Discussion**. The resulting immunogenicity was lower than that observed in clinical trials, probably due to the presence of immune disease and immunosuppressive treatment, and to the fact that this was a real-world study. No differences in response according to age, previous virus zoster reactivation, or concomitant treatments were found. **Conclusions**. RZV was well tolerated and reached the immune response objective in 40% of patients. These results reinforce the importance of including RZV vaccination for immunosuppressed patients. Real-world studies regarding vaccine effectiveness are still needed in order to gain a full understanding of the response to RZV in this group of patients.

## 1. Introduction

Immune-mediated inflammatory diseases (IMIDs) are chronic entities of unknown etiology, characterized by anomalous autoimmune responses and resulting in tissue and organ damage. Their treatment is based on corticosteroids and immunosuppression. Immunosuppressive therapy based on disease-modifying antirheumatic drugs (DMARDs) can be divided into the following categories: conventional synthetic drugs (csDMARDs), such as methotrexate, leflunomide, sulfasalazine, and hydroxychloroquine; biological drugs (bDAMRDs), such as anti-TNF, anti-IL-6, anti-IL-17, and anti-IL 13/23; targeted synthetic drugs (tsDMARDs), such as apremilast and Janus kinase inhibitors (JAKis), among others [1]. The Janus kinase–signal transducer and activator of transcription (JAK/STAT) family of proteins constitute transmembrane cell receptors, which send signals from extracellular cytokines to intracellular ones and activate gene expression. The JAK/STAT pathway is crucial in many pathogenic immune system dysregulations, and it has been suggested that its inhibition would prevent aberrant immune responses [2,3]. Four oral JAKis are approved for IMIDs. Upadacitinib and filgotinib are selective for JAK-1, while baricitinib is selective for JAK-1 and JAK-2. Tofacitinib is a panJAK inhibitor for JAK-1, JAK-3, and, to some extent, JAK-2 [1]. Patients with IMIDs are known to present a higher risk of infections than healthy individuals, due to the disease itself and the need for immunosuppressive drugs [4,5]. Innate, humoral, and cellular immunity are affected by IMIDs and DMARDs; hence, worldwide scientific societies have approved specific vaccine recommendations for this selected group of patients [5]. JAKi treatment showed a similar risk for bacterial infections when compared with bDMARDs, but a higher risk for viral infections was observed, especially for varicella zoster virus (VZV) reactivation. The exact incidence is not well determined, and it could depend on the type and dose of JAKi treatment. The risk might also increase when JAK-2 or JAK-3 are inhibited. The reactivation of VZV is the most recognized infectious complication of JAKi treatment [6,7].

VZV is a neurotropic alpha-herpes virus, transmitted from person to person via inhalation or contact. In its primoinfection, it causes chickenpox, which mainly occurs in children, with an incidence of almost 100% in some regions of the world. The virus persists throughout life, remaining latent in the sensory ganglia, and its reactivation causes herpes zoster disease, also called shingles, years after initial infection. Herpes zoster occurs in 30% of the population at some point in life, with 3–5% subsequently experiencing recurrences [8,9]. Shingles symptoms consist of painful and itchy vesicular lesions on an erythematous basis, which are typically distributed according to a dermatome. Shingles occurrence is followed by post-herpetic neuralgia as a main complication in 18% of the patients. Other possible sequelae include structural damage if affecting the eye, neurological involvement, or exacerbation of cardiological diseases, resulting in long-term burdens and deeply affecting quality of life. Risk factors for zoster reactivation and zoster complications include elderly age and immunosuppressive therapies [10]. The innate host immune response to viral infection is dominated by the transcription of interferons (IFNs), induced by pattern recognition receptors (PRRs) and associated molecular patterns (PAMPs). INFs activate innate cells, such as natural killers, and the JAK/STAT pathway, resulting in the expression of multiple proteins that inhibit virus growth. In addition, IFNs activate the adaptive immune response by stimulating dendritic cell maturation and antigen presentation. The VZV uses multiple evasion strategies to inhibit IFN signal transduction via the JAK/STAT pathway and minimize the antiviral response. This could be the reason why there is a high risk of reactivation and severity in patients treated with JAKis [6,11].

On the basis of previous data about the severity of HZ infection in IMID patients, vaccination against HZ has been increasingly encouraged. A live-attenuated VZV vaccine (ZVL (Zostavax, Merck Sharp & Dohme, Rahway, NJ, USA)) and an adjuvanted recombinant zoster vaccine (RZV (Shingrix, GSK)) are licensed for the prevention of HZ in adults ≥50 years of age.

ZVL was approved in 2006 and showed a vaccine effectiveness (VE), measured as the reduction in zoster reactivation incidence, of 50% in patients older than 60 years, and 70% in patients between the ages of 50 and 59 years [12,13]. However, ZVL is not recommended in patients with compromised immunity (from disease or treatment), as live-virus vaccines can cause severe or even fatal reactions in this group of patients, due to uncontrolled replication of the vaccine virus. RZV is a recombinant subunit herpes zoster vaccine that was licensed in Europe in 2021 for patients ≥50 years of age, showing a clinically acceptable safety profile, with a VE of 97.2% in patients older than 50 years [13], and 89.8% in patients older than 70 years [14]. It is administered in two doses, containing VZV glycoprotein E (gE), which directs the immune response to the virus itself as a vaccine antigen, and the AS01B adjuvant system, to stimulate T-cell immunity to recombinant proteins [15]. This vaccine is suitable in immunocompromised patients, and it has recently become available in Spain for patients treated with JAKis, among other indications. Although there are clinical trials on patients suffering from hematologic malignancies, solid tumors, renal transplants, and human immunodeficiency virus (HIV), no clinical trials were performed in patients with IMIDs, nor in patients on JAKi treatment. In addition, there is a lack of real-world effectiveness, immunogenicity, and safety studies in this group of patients.

Therefore, we decided to evaluate the immunogenicity and safety of RZV administered to IMID patients treated with JAKis in a prospective study.

## 2. Materials and Methods

### 2.1. Study Design

This was a real-world, observational–descriptive, retrospective, transversal and single-center cohort study, conducted in a hospital of Madrid. All the investigators acted in accordance with the principles of the Declaration of Helsinki. Written consent was obtained from all participants. The study was approved by the appropriate institutional review boards (Comité regional de la Comunidad de Madrid: EC 43.22).

### 2.2. Inclusion and Exclusion Criteria/Participants

Patients with IMIDs on JAKi treatment who were admitted to the rheumatic department of Infanta Sofía University Hospital, Madrid, Spain, were recruited for this study. The recruitment period occurred between 1 June 2022 and 28 February 2023. All patients included in the study fulfilled the inclusion criteria.

Patients aged ≥18 years, followed for IMIDs such as rheumatoid arthritis (RA), psoriatic arthritis (PsA), spondylarthritis (SpA), and systemic erythematosus lupus (SLE), over at least 4 weeks of treatment with JAKi (upadacitinib, baricitinib, filgotinib, or tofacitinib) were enrolled in the study. Concomitant treatment with csDMARDs (methotrexate, leflunomide, sulfasalazine, or hydroxychloroquine) or corticosteroids, up to 5 mg of prednisone or equivalent per day, was permitted. We excluded patients who had received other VZV vaccinations in the last five years. Patients who interrupted the JAKi treatment during the period of vaccination, or who did not complete vaccination, were also excluded from the study.

### 2.3. Procedures

Data regarding gender, age, previous HZ infection, duration of IMIDs, the start date of JAKi treatment, the type of JAKi, and concomitant treatments were collected. Participants received two intramuscular deltoid injections (0.5 mL per each dose) of the RZV vaccine, 2 months apart. The vaccine was supplied as a vial of the lyophilized recombinant VZV surface gE antigen component, which was reconstituted at the time of use with the accompanying vial of the AS01B adjuvant suspension component. Nine milliliters of blood in lithium heparine were sampled prior to the first vaccine dose and 1 month after the second one.

### 2.4. Objectives and Outcomes

Humoral immune response was tested through a chemiluminescence immunoassay (CLIA), using varicella zoster virus gE as the antigen against which to measure anti-gE levels. The CLIA kit used was the Liaison VZV IgG (LIAISON^®^ VZV IgG) (REF 310850) by DiaSorin S.p.A. (Saluggia, Italy). varicella-zoster virus antigen is used for coating magnetic particles (solid phase) and a mouse monoclonal antibody to human IgG is linked to an isoluminol derivative (isoluminol-antibody conjugate). During the first incubation, varicella-zoster virus antibodies present in samples bind to the solid phase. During the second incubation, the antibody conjugate reacts with varicella-zoster virus IgG already bound to the solid phase. After each incubation, the unbound material is removed with a wash cycle. Subsequently, the starter reagents are added and a flash chemiluminescence reaction is thus induced. The light signal, and hence the amount of isoluminol-antibody conjugate, is measured by a photomultiplier as relative light units (RLUs) and is indicative of the varicella-zoster virus IgG concentration present in samples. An antibody concentration higher than 165 µg/mL was considered positive. For initially seropositive patients, the cutoff for the vaccine response assessment was an antibody concentration that was, at the second measurement, four or more times the pre-vaccination value, according to the setting of the clinical trial NCT01165177. We also assessed the influence on the humoral response to the vaccine of several factors such as age, previous zoster reactivation, time since IMIDs diagnosis, time on JAKi, and concomitant treatment (csDMARDs or corticosteroids).

Safety was evaluated in the medical visits following vaccination. Adverse effects were considered when they appeared until 1 month after each vaccine dose, according to the setting of the clinical trial NCT01165177. They were divided into mild local (pain and/or redness), mild systemic (fever, myalgia, fatigue), severe local (cellulitis), and severe systemic (cardiovascular, pulmonary, or nervous system complications). However, severe systemic adverse effects were recorded from first vaccination until the end of the study (one month after second vaccination). Disease flare was defined as the appearance of articular pain and swelling, an increase in C reactive protein (CRP), and high scores in the following disease evaluations: Disease Activity Score-28 (DAS-28), Clinical Disease Activity Index (CDAI), Bath Ankylosing Spondylitis Disease Activity Index (BASDAI), Ankylosing Spondylitis Disease Activity Score (ASDAS), Disease Activity Index for Psoriatic Arthritis (DAPSA), and SLE Disease Activity Index (SELENA-SLEDAI) for SLE.

### 2.5. Statistical Analysis

For descriptive analysis, absolute (n) and relative (%) frequencies were used to express the qualitative variables, and the mean ± standard deviation (SD) or the median and interquartile range (Q1–Q3) were used to express the quantitative variables, based on parametric behavior.

Data analysis was performed with SPSS version 29.0 software (IBM Corp; Armonk, NY, USA).

## 3. Results

In total, 49 patients were included in the study, 37 of whom were women (75.5%), with a mean age of 58.2 ± 11.0 years. The main diagnosis was RA, present in 36 patients (73.5%), followed by 7 with PsA (14.3%), 5 with SpA (10.2%), and 1 with SLE (2.0%). The median time of diagnosis expressed in years was 8 [Q1–Q3 4.5–15.0]. A total of 27 patients (55.1%) were on treatment with upadacitinib, along with 15 (30.6%) on baricitinib, 4 (8.2%) on filgotinib, and 3 (6.1%) on tofacitinib. The median time on JAKi treatment, expressed in months, was 9 [Q1–Q3 4.0–19.0]. A total of 33 (67.4%) patients had concomitant csDMARD treatment. Of them, nine (56.3%) were on methotrexate and seven (43.7%) were on leflunomide. A total of 17 (34.7%) patients were under low-dose prednisone treatment. Six (12.2%) reported previous clinical HZ infection. None of the patients had previously received the ZVL live vaccine. At the first vaccination, 31 patients were at remission, while 8 and 10 patients presented mild and moderate disease activity, relatively. No patient was vaccinated presenting a high disease activity. The main demographic characteristics are presented in Table 1.

**Figure 1:**
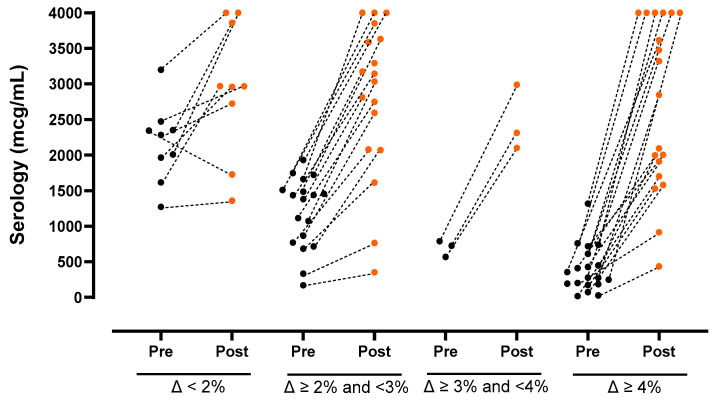
Evaluation of humoral vaccine response to RZV in IMIDs patients under JAKi treatment. The figure shows the increase in anti gE VZV antibody concentrations, measured with CLIA, comparing the initial antibody concentration (black dots) and the post-vaccination (orange dots) serology in serum.

At the pre-vaccination measurement, the median anti-VZV IgG serum concentration was 770 (345–1562) µg/mL, with no differences between the group with previous HZ reactivation and the group without. The seroprevalence was 93.9%, and only three patients (6.1%) had a negative anti-VZV concentration. At the post-vaccination measurement, 1 month after the second vaccine dose, the median anti-VZV concentration increased to 2968 (2002–3853) µg/mL. We found that 19 patients (38.8%) reached the immune response objective, quadrupling their initial antibody concentration in the post-vaccination serology, compared with the pre-vaccination one. The concentration was at least tripled in 22 patients (44.9%), and at least doubled in 40 (81.6%) (Figure 1). The median increase in antibody concentration in the second serology was 2.8 times the pre-vaccination one. Factors that could have influenced the vaccine response, such as age, report of previous HZ reactivation, concomitant treatment with low-dose corticosteroids or csDMARDs, time since IMIDs diagnosis, or time since JAKi was initiated, were also studied. All these factors showed either no statistical significance or no association whatsoever with the serological response. All patients (100%) who had a negative initial basal antibody concentration reached a positive concentration after the second dose. Regarding the 19 patients who reached the immune response objective (16 females/3 males), 14 had an RA diagnosis, 2 had PsA, another 2 SpA, and the remaining one SLE. Furthermore, 11 patients were on upadacitinib, six on baricitinib, one on tofacitinib, and one on filgotinib. Additionally, four patients were on methotrexate and three on leflunomide. Seven patients were on low dose prednisone. Three out of the 19 patients presented an initially negative serology.

A total of 17 (34.7%) and 15 (30.6%) patients reported adverse effects after the first and second vaccinations, respectively. All of them except one were mild; 19 (59.4%) consisted of injection site pain, and 11 (34.4%) were systemic, such as myalgia, fatigue, or fever. The only severe adverse effect was local, consisting of swelling that required 5 days of antibiotic treatment. One patient suffered from a hemorrhagic stroke 34 days after the first vaccination dose. He was a 65-year-old male, obese and an active smoker, diagnosed with hypertension and under two anti-hypertensive treatments, as well as anticoagulant treatment due to an auricular fibrillation. After analyzing this medical history, it is logical to assume that the etiology of the stroke could likely be attributed to the pre-existing cardiovascular factors and anticoagulant treatment, rather than to the vaccine. Regarding the group patients who reached the vaccine response objective, four suffered of mild local adverse effects and another four of mild systemic adverse effects. No other severe systemic adverse effects were reported. Three patients presented a disease flare in the 4 weeks following vaccination that required a corticosteroid cycle, and none of them needed a base medication dosage increase.

## 4. Discussion

One in three people will develop herpes zoster in their lifetime, a likelihood that increases when regarding IMIDs patients and JAKi treatment. The goal of this study was to evaluate the immunogenicity and safety of two doses of the RZV vaccine in IMIDs patients treated with JAKi, as recommended by different international and national organizations. To the best of our knowledge, this is the largest real-world cohort study published on this topic.

The antibody titer measurement technique available in our hospital, as well as in many other centers around the world, is the CLIA test. In the main clinical trials, the ELISA method was used for antibody measurement. Some studies showed that the CLIA test is an alternative method that meets the quality requirements and has an equivalence of 95.8%, with a higher sensibility and specificity when compared with ELISA [16].

In terms of immunogenicity, slightly more than one-third of the patients (38.8%) reached the immune response objective established. In clinical trials, with non-rheumatic immunosuppressed patients, the humoral immune response ranged between 65% and 96% [17,18], and, in real-life studies with a healthy cohort, the immunogenicity achievement was between 70% and 80%. There is a lack of real-life studies analyzing, in the same terms as in the clinical trials, the immunogenicity acquired by immunosuppressed patients. Therefore, the difference in immunogenicity achievement could be explained by the fact that this was a real-life study including patients with immune disease under immunosuppressive treatment. Indeed, when the basal antibody concentrations are already high, it is more difficult to reach a fourfold immune response than when the initial concentrations are low, because, in the first situation, the immune system is already activated and more quickly controls the incitement caused by a virus or a vaccine. The prevention of HZ reactivation was not included as an intended clinical outcome of this study.

We attempted to evaluate differences in immune response depending on age, previous HZ reactivation, time since IMIDs diagnosis, concomitant treatment with low-dose corticosteroids or csDMARDs, type of JAKi treatment, and time on JAKi treatment. However, it is probable that the sample was too small to draw any conclusion, as no statistically significant difference was found. It is important to highlight that all patients who initially had a negative antibody level achieved a positive level after vaccination.

Regarding adverse effects, most patients tolerated the RZV vaccine well, with only a few cases of mild adverse effects and one local severe reaction. Although vascular events related to vaccines are usually prothrombotic, hemorrhage has also been described in this context. The physiopathological reason is, at the moment, not well described, yet some theories point toward platelet variations [19,20]. However, the cardiovascular risk presented by our patient, as well as previous anticoagulant therapy, suggested that the hemorrhagic stroke and the RVZ vaccine were very unlikely to be linked. Furthermore, a recent study highlighted a higher stroke risk after herpes zoster infection, and they observed a decrease in this risk after RZV vaccination [21]. Regarding disease activity, only a few patients presented a disease flare after vaccination, an incident that is common with other vaccines, such as the COVID19 vaccine, probably owing to immune system overactivation [22]. None of them required treatment escalation or hospitalization.

Vaccination is the best strategy through which to induce protection in IMIDs patients receiving immunosuppressive treatments. Worldwide, multiple vaccines are recommended and highly demonstrate a lower incidence of infections. Unfortunately, until very recently, there was no available non-live vaccine appropriate for immunosuppressed patients that provided protection against reactivation of VZV, which, albeit usually not a life-threating illness, can induce tiresome chronic physical sequelae. Since RZV became available, many physicians began including it in the vaccine schedules of their immunosuppressive patients, with the hope of prevention.

There were limitations to our study, as there were no controls, and the sample size was small. Furthermore, there was no evaluation of the cellular immune response since it is not always available in real-world studies and standard clinical centers. However, it would be beneficial to include it in future research when available. In addition, we cannot identify whether the adjuvant or the gE protein itself caused the adverse events experienced by the patients. Despite these limitations, our results might help to highlight the situation for this concrete group of patients with a high risk of VZV reactivation, as well as the potential adverse effects of the RZV vaccine.

## 5. Conclusions

Rheumatic patients under immunosuppressive treatments are at high risk of infection. Our study showed that RZV was immunogenic in patients with AIIRDs treated with JAKis, but at lower levels than those observed in the clinical trials. The vaccine was well tolerated. These results reinforce the importance of including RZV vaccination in immunosuppressed patients.

## Figures and Tables

**Table 1:** General characteristics and descriptive analysis of participants.

	Total
	(n = 49)
**Age, years**	58.2 ± 11.0
**Gender, n (%)**	
Male	12 (24.5)
Female	37 (75.5)
**Previous HZ, n (%)**	
No	43 (87.8)
Yes	6 (12.2)
**Diagnosis, n (%)**	
RA	36 (73.5)
PsA	7 (14.3)
SpA	5 (10.2)
SLE	1 (2.0)
**Time since diagnosis, years**	8 [4.5–15.0]
**Corticosteroids, n (%)**	
No	32 (65.3)
Yes	17 (34.7)
**csDMARDs, n (%)**	
No	33 (67.3)
Yes	16 (32.7)
**Types of csDMARDs, n (%)**	
MTX	9 (56.3)
LFN	7 (43.7)
**JAKi, n (%)**	
Upadacitinib	27 (55.1)
Filgotinib	4 (8.2)
Tofacitinib	3 (6.1)
Baricitinib	15 (30.6)
**Time on JAKi, months**	9 [4.0–19.0]
**Disease activity n (%)**	
Remission	31 (63.3)
Mild	8 (16.3)
Moderate	10 (20.4)
High	0 (0)

Qualitative variables expressed as absolute (n) and relative (%) frequencies. Age statistics show mean± standard deviation, while the other quantitative variables express median and [Q1–Q3].

## Data Availability

Not applicable.
